# Fruits as Prospective Reserves of bioactive Compounds: A Review

**DOI:** 10.1007/s13659-018-0186-6

**Published:** 2018-08-01

**Authors:** Marines Marli Gniech Karasawa, Chakravarthi Mohan

**Affiliations:** 1 Department of Agriculture and Forestry, Palermo University, Palermo, Italy; 2 Department of Genetics and Evolution, Federal University of São Carlos, São Carlos, SP Brazil

**Keywords:** Anticancer, Antioxidant, Bioactive compounds, Flavones, Fruits, Medicinal uses

## Abstract

Bioactive natural products have always played a significant role as novel therapeutical agents irrespective of their source of origin. They have a profound effect on human health by both direct and indirect means and also possess immense medicinal properties. Fruit species are largely appreciated and highly consumed throughout the world. Epidemiologic information supports the association between high intake of fruits and low risk of chronic diseases. There are several biological reasons why the consumption of fruits might reduce or prevent chronic diseases. Fruits are rich sources of nutrients and energy, have vitamins, minerals, fiber and numerous other classes of biologically active compounds. Moreover, parts of the fruit crops like fruit peels, leaves and barks also possess medicinal properties and have been included in this review. The most important activities discussed in this review include antidiabetic, anticancer, antihypertensive, neuroprotective, anti-inflammatory, antioxidant, antimicrobial, antiviral, stimulation of the immune system, cell detoxification, cholesterol synthesis, anticonvulsant and their ability to lower blood pressure. Several phytochemicals involved in this context are described with special emphasis on their structural properties and their relativity with human diseases.

## Introduction

Since birth, several adverse factors affect human health. Bioactive compounds are present in small quantities in foods and their effect on health is being continuously investigated. The epidemiologic information centre points out that high intake of fruits will invariably lead to reducing the risk of chronic disease. Moreover, parts such as bark, leaf, flower and roots also possess beneficial properties to humans. In fact, some wild fruit species like *Psychotria* genus, are not normally consumed by man but their parts have numerous pharmaceutical and medicinal properties [1, 2].

Citrus fruits and limonoids were indicated to act by preventing heart disease, inflammation and arterosclerosis with their hepatoprotective, antimicrobial, neuroprotective, antioxidants, anti-diabetic properties and also play a pivotal role against several cancers [3–6]. Pomegranate is also been reported to contain medicinal properties against several types of cancers [7, 8]. The extract of fruits of *Phaleria macrocarpa* regulates hormonal imbalance in women and acts against dysmenorrhoea, endometriosis and cancer [9]. In addition, fruit extracts of *Thevetia peruviana* have shown potential activity against cancer [10]. In vitro and in vivo pharmacological studies of *Passiflora edulis* have disclosed antioxidant, antifungal, antitumor, anti-inflammatory, anti-anxiety, and antihypertensive activities [11]. *Passiflora nitida* is reported as having antioxidant, anti-inflammatory and hypoglycaemic effects [12]. Berry fruits have several bioactive compounds that have antioxidant, anti-inflammatory, cardioprotective and neuroprotective effects [13]. *Terminalia* species were reported as having produced effective results in controlling cancer [14]. Leaf extracts of *Persea americana* (avocado) have shown anti-hypoglyceamic with anti-diabetic effect [15, 16], vasorelaxant and anticonvulsant activity [17], while the avocado seed extract is considered as a good source of antioxidant [18], protects against cardiovascular disease, has hematopoietic, antimicrobial, anti-viral, and anti-inflammatory activities, anti-arthritic and anticancer effects, and helps to control body weight, while fruit extracts have shown to possess hepatoprotective effect [17]. Ethanolic extracts of *Osmanthus matsumuranus* have been proven to be effective against three types of cancers [19]. Recently antihyperglycemic [20] and antioxidant activities in the Brazilian native species *Psidium cattleianum*, *Butia odorata* and *Eugenia uniflora* have been reported [21]. *Eugenia uniflora* also produced antimicrobial [22], anti-depressant and anti-obese activity [23]. The antidiarrheal effect of leaf and fruit extracts of the Brazilian fruit tree *Plinia cauliflora* has been reported [24]. The antioxidant activity in leaves of the following mediterranean fruit species: cherry, peach, olive, plum, pear, apple, chestnut and pistachio were investigated and the results demonstrated that they offer powerful sources of bioactive phenol compounds for pharmaceutical purposes, beverages and natural pesticides [25]. In vitro and in vivo anti-diabetic activity of leaf and fruit extracts of *Juniperus* was reported [26]. Fruit extract of *Xylopia aethiopica* has proven to contain antioxidant and anticancer activity [27]. The juice extract of *Syzygium cumini* (also known as *Eugenia jambolanum*) is yet another fruit species which was observed to possess anti-inflamatory, anti-diabetic, antibacterial and gastroprotective activities [28]. *Eugenia dysenterica* (also known as cagaita) encapsulated fruit extract was reported to have antimicrobial and antioxidant activity [29].

Investigation on *Plinia cauliflora* leaf extract verified that the major bioactive compound producing antifungal effect was the hydrolysable tannin casuarin [30]. Antioxidant and nutritional properties of six tropical fruits were investigated [31] and gallic acid was determined to be the most abundant antioxidant among the six fruit species. Pharmaceutical and medical interest is increasing over *Psychotria* genus which are rich in alkaloids (like: emetine, cephalin, dimetiltriptamin), coumarins, terpenoids, flavonoids, tannins and cyclic peptides [32]. Benzyl glucosinolate obtained from *Carica papaya* immature fruits and seeds was reported to have anticancer effect [33]. *Physalis* was found to be possessing higher carotenoid content [34]. In *P. americana* (avocado) the chemical compounds reported in seed indicate the presence of flavonoids, alkaloids, saponins, esteroids, tannins and sterols [17]. *Olea europea* was reported to have abundant bioactive compounds showing in vivo and in vitro antioxidant, anti-diabetic, anti-inflammatory, anti-convulsant, antimicrobial, antiviral, analgesic, immunomodulatory, antihypertensive, anti-hyperglycaemic, anticancer, antinociceptive, gastroprotective and wound healing actions [35]. In this context, this review summarizes the various bioactive compounds that contribute to the medicinal properties of different fruit species. In addition, structure activity relationships of fruit bioactive compounds are described. This review also highlights the novel approaches that could be utilized for the complete exploitation of fruit polyphenols.

## Fruits Species and Their Medicinal Properties: An Update

Fruits possess an array of medicinal properties and act against several human diseases owing to which they are highly consumed worldwide. The various medicinal properties of fruits and the bioactive compounds associated with them are described in this section with examples.

### Antioxidants

A majority of fruit species are rich in antioxidants. Recently, the changes in the phenolic compounds of eight fruit species was evaluated and significant differences between the antioxidant and phenolic content were recorded [36] and the results showed that *Aronia melanocarpa* and *Sambucus nigra* were good sources of antioxidant properties while highest antioxidant activity was recorded in *Vaccinium myrtillus*. Hydro-alcoholic leaf extracts of *P. edulis* and *P. nitida* have shown strong antioxidant effect in in vivo and in vitro tests [11, 12]. The extract of native fruits like *Psidium cattleianum*, *Butia odorata* and *Eugenia uniflora* was evaluated in in vitro tests by [21] verifying powerful antioxidant action due to the presence and combination of phenolic compounds, anthocyanins, carotenoids and reducing sugars was observed. Citrus spp. is claimed to be having antioxidant activity due to the predominance of bioactive compounds found in this species [3–6], but it was verified that the antioxidant activity was higher in the peel than in the fresh tissues [37]. The antioxidant properties of *P. americana* seeds were reported [17, 18]. Six tropical fruit species were evaluated [31] to decipher the antioxidant activity and the results determined that the chemical compounds in banana and lichti were epigallactochin and quercetin, in pineapple and mango were ferrulic, sinapic, syringic and gallic acids, and in passion fruit was piceatannol. The antioxidant activity was investigated in aqueous methanol extract of leaves of *Prunus avium*, *Prunus persica*, *Prunus domestica*, *Olea europea*, *Pirus communis*, *Pirus malus*, *Pistachia verra*, and *Castanea sativa* and it was found that the leaves of chestnut and plum have the greatest antioxidant activity while lowest was observed in peach, olive and cherry [25]. *Psychotria* genus is cited as possessing highl anti-oxidant activity which might be due to the presence of monoterpene alkaloids psycolatine, brachicerine isolated from *P. brachiceras* and *P. umbellata* [2]. Chokeberry and elderberry were also indicated as good sources of antioxidant [36]. Wounding induced higher antioxidant activity in pitaya fruits [38]. Antioxidant activity was also verified in essential oils obtained from fruit extract of *Xylopia aethiopica* [27]. High antioxidant activity was also observed in *Aronia melanocarpa* fruits [39]. Encapsulated fruit extract of *Eugenia dysenterica* also have been reported to have antioxidant activity [29]. Olive is also reported to produce antioxidant activity by leaf ethanolic extract, aqueous extract and infusion, fruit and leaves, pulp [35]. *Euterpe edulis* or Juçara fruit, found mainly in southern and south-eastern regions of Brazil, is rich reservoir of bioactive compounds such as anthocyanins, flavonoids and phenolic acids and its antioxidant potential has been widely investigated [88]. Mulberry fruits are also abundant in bioactive compounds that showed high in vitro antioxidant capacity, apart from other medical uses [89].

### Anticonvulsant

Anticonvulsant activity was studied by [40] in extracts from leaves of the Ukrainian flora shrubs showing that *Corylus avellana* dry aqueous extract can be a promising substance with anticonvulsant properties. Avocado leaf extract also was reported having anticonvulsant activity [17]. Olives were also reported having anticonvulsant properties [35].

### Anticancer

Anticancer effect is reported in the review done by [7] with the fruit of *Punica granatum* showing in vivo and in vitro results obtained by several authors for breast cancer, prostate cancer, colorectal cancer, leukemia, glioblastoma and hepatocellular carcinoma obtained in animal and human clinical trials. Pomegranate was shown to inhibit lung, prostate and urinary bladder urothelial carcinoma by ethanol extract and juice [8]. In the same way, some reviews have shown that *Citrus* species have bioactive compounds that can act against different types of cancer being cited as narigin and hesperidin [4, 5] with positive results against lung, colon and breast cancers [3]. Also, the citrus limonoids chemical compounds limonin, nomilin, deacetylnomilinic acid, isolimonelic acid, nominilic acid are described as having anticancer effect [5]. *Phaleria macrocarpa* fruit extracts are reported to be effective against breast and cervical cancers [9]. The effect of adamantyl derivatives and rearranged benzophenones obtained from fruit extracts of *Garcinia xanthochymus* was investigated by [41] that found powerful inhibitory activity against four cell lines of human cancer. Also, [10] studied the anticancer potential of *T. peruviana* and verified that the methanolic extract of this species showed cytotoxic effect against cell lines of colorectal, breast, prostate and lung cancer. Anti-tumor activity was also tested in *P. edulis* fruit decoction being observed inhibitory action over the metalloproteases MMP-2 and MMP-9 that are involved in the tumorogenesis [11]. Still, methanolic extracts of Terminalia were effective against breast cancer and *T. bellerica* was better against liver and colon cancers, but for *T. laxiflora* no effect was observed [14]. Anticancer activity was also investigated with *Osmanthus matsumuranus* being observed that methanolic extract act over hepatocellular carcinoma, colon and lung carcinoma by suppressing cell proliferation, inducing apoptosis and cell cycle arrest of G2/M phase [19]. Another plant described as having anticancer activity is *Psorela corylifolia*, and the ethanolic extract of dried and ripe fruits was tested in human colon cancer lines being observed the suppression of growth and decrease in the expression of the protein cyclin D1 and CDK4 [42]. Another tropical fruit species with anticancer properties is *Eugenia jambolana*. The extracts of fruits have produced effect against colon cancer by cell apoptosis [43]. Essential oils obtained from fruit extract of *Xylopia aethiopica* exert antiproliferative effect against human cervical cancer and prostate cancer [27]. There are some evidences that *Aronia melanocarpa* fruits also prevent cancer disease in human [39] Benzyl glucosinolate obtained from *Carica papaya* seeds and immature fruits also produced some effect against lung cancer lines [33]. Flavonoids, carotenoids and persin found of *P. americana* seeds have been reported having anticancer properties [17]. Extract obtained from *Olea europea* leaves were reported to have several chemical compounds acting against colon cancer, breast cancer, adenocarcinoma, leukemia, and cervical carcinoma [35].

### Antimicrobial

Bacterial diseases are very common in humans and are considered critical at times. Antibacterial activity was evaluated in *C. sinensis* (sweet orange) which proved that the cultivars *Sisila*, *BAN* and *MT* possessed anti-bacterial activities against *Escherichia coli* and *Staphylococcus aureus*; the cultivar *Biblia sweet and the sour orange C. aurantium* prompted strongest effect against *E. coli* and *Staphylococcus aureus*, and MRSA [44], but seed oil was shown to have better effect than fruit peel against *Staphylococcus aureus* and *Candida albicans* [45]. Moreover, citrus chemical compounds like limonin, nomilin, obacunone, inchanging and isoobacunoic acid are pointed out acting against several microbial species, like virus, bacterial, fungal [3–5] and larvicidal [5]. In vitro studies with peptides purified from *P. edulis* seeds were active against *Trichoderma harzinum*, *Fusarium oxysporum* and *Aspergillus fumigatum* [11]. *Plinia cauliflora* leaf extracts have proven to be effective in management of five different *Candida* species [30] and are also active against *Enterobacterium fecalis*, *E. coli*, *Salmonella* and *S. sonei* [24]. Anti-microbial activity was also reported in the *Psychotria* genus with klugin, cephalin, and isocephalin being the chemical compounds responsible for controlling *Leishmania donovani* and *Plasmodium falciparum*, and 14-oxoprunifoleine and stricosamide responsible for controlling *Leishmania amazonensis.* Leaf extracts of Eugenia uniflora proved efficient against *Leishmania amazonensis* [22]. Antiviral activity was reported with *P. ipecacuanha* alkaloids and emetine was found to inhibit the HIV virus, while *P. serpens* extract suppressed herpes simplex [2]. *Syzygium cumini* fruit juice extract possessed antibacterial and gastroprotective activity [28]. In order to offer alternative to control childrens’ diarrhoea fruit and leaf extract of *Plinia cauliflora* was investigated [24] and it was found that leaf and fruit ethanolic extract was more effective than ampicillin against *Enterobacterium fecalis*, and leaf extract produced better effects than ampicillin against *E. coli* and *Salmonella* sp. but not against *S. sonei*. Anti-bacterial and anti-viral effect was also observed in *Aronia melanocarpa* [39]. Encapsulated fruit extract of the Brazilian native plant *Eugenia dysenterica* was reported to have produced antimicrobial activity against *Staphylococcus aureus* and *Lysteria monocytogenes* [29]. Avocado has been reported having antimicrobial activity acting against *Plasmodium falciparum*, *Heliobacter pillory*, and herpes simplex tipe1virus, human immunodeficiency virus1 and adenovirus [17]. Olive leaf extract was reported to produce inhibitory activity against *Heliobacter pillory*, *Campylobacter jejuni*, and *Staphylococcus aureus*, *Bacillus cereus*, *B. subtillis*, *Pseudomonas aeruginosa, Escherichia coli*, *Klebsiella pneumonia*, *Candida albicans*, *Cryptococcus neoformans*, *Salmonella typhimurium*, *Lysteria monocytogenes*; the acetone extract of leaves also inhibited *Salmonella enteiritidis*, *Streptococcus thermophiles*, *Enterococcus fecalis*, and *Lactobacillus bulgaricus* [35].

### Effect Against Cardiovascular Diseases

Cardiovascular diseases are one of the biggest problems in human health. To control this disease some reports have shown that *P. edulis* methanol extracts [11] and pulp fruit were able to lower blood pressure in hypertensive rats [46]. Also, *Eugenia uniflora* leaf extracts were reported as being effective in lowering blood pressure in hypertensive rats [20]. The protection of cardiovascular disease was also reported in *Aronia melanocarpa* [39]. *P. Aamericana* seed extract was reported to protect cardiovascular diseases and the fruit extract produced hepatoprotective effect [17]. Olives have also been reported as having anti-hypertensive and cardio-protective activity as is evident by the chemical compounds uvaol, ursolic acid and oleanolic acid isolated from the leaves [35].

### Against Inflammation

Inflammatory genes related to endometriosis were investigated with the bioactive compound DLBS 1442 obtained from fruit extracts of *P. macrocarpa* and found that it increased the cells in sub-G1 stage and exhibited inhibitory effect on proliferation, migration and angiogenesis [9]. Anti-neuroinflammatory effect was found in *Citrus reticulate* peels by [4] being indicated that the most chemical compounds that show anti-inflammatory effects are: limonin, nomilin and citrusin [5]. Also, anti-inflammatory effect was detected in of *P. edulis* and *P. nitida* aqueous leaf extract with powerful effect in vivo, and in systemic administration being effective in suppressing tumour necrosis [11, 12]. Another plant with anti-inflammatory effects is *Prunus persica* [47]. Extract of the *Psychotria* species were evaluated in vitro being the most pronounced anti-inflammatory activity found in *P. suturela*, *P. stachioides* and *P. capitata* [2]. Anti-inflammatory effect was also observed in the juice extract of *Syzygium cumini* (also known as *Eugenia jambolanum*) [28]. *Aronia melanocarpa* seems also have anti-inflammatory effect [39]. Another species, *Persea americana* hematopoietic also was reported as having anti-inflammatory activity [17]. High anti-inflammatory activity by Olive oil and *n*-Hexane extract of fruits was reported in rats [35].

### Against Diabetes

Anti-diabetic effect is found in several plant species. Hydro-alcoholic leaf extracts of *P. edulis* and *P. nitida* were dynamic in the control of diabetes [11, 12]. Leaf extracts of *P. americana* produced anti-hyperglyceamic effect in Wistar rats [15, 17] and also anti-diabetic effect [16]. On the other hand, the ethnobotanical use of 50 different species to control diabetes was evaluated [48] and among them nine species were the most cited (*Giuburtia tessmannii*, *Anonna bonei*, *Carica papaya*, *P. americana*, *Anonna muricata*, *Ceiba pentandra*, *Coccos nucifera*, and *Piriclima nitida*), with the majority of them using stem bark (50%), leaves (26%), and other parts (24%). In vitro test using the extract of Brazilian native fruits *Psidium cattleianum* (araça), *Butia odorata* (butia) and *Eugenia uniflora* (pitanga) were indicated as having bioactive compounds that decrease blood glucose and defend patients with type II diabetes [21]. Citrus nomilin was the chemical compound identified of having anti-diabetic activity [5]. Leaf and fruit extracts of *Juniperus foetidissima* and *Juniperus sabina* were tested in vitro and in vivo against diabetes inhibitory effect by the major chemical compound identified as ametoflavone was observed [26]. *Syzygium cumini* fruit extract produced anti-diabetic activity [28]. The prevention of this chronic disease was also described for *Aronia melanocarpa* [39]. Anti-diabetic effect was reported being achieved in *Olea europea* leaves aqueous and ethanolic extract, oleanic acid, oleuropein and hydroxytirosol for in vivo and in vitro tests [35].

### Effect Against Other Diseases

Several other diseases/disorders affect humans in the modern society. Majority of population are affected with obesity, anxiety and depression to name a few. Leaf extract of *Eugenia uniflora* was evaluated in mice and produced anti-obesity effect [23]. *P. edulis* aqueous extract has proven be a natural anti-depressant and anti-anxiety activity [11]. *Eugenia uniflora* leaf extract also acted as anti-depressant in mice [23]. The juice extract of *Syzygium cumini* [28] and *Aronia melanocarpa* have produced gastroprotective effect [39]. Seed extract of *P. americana* have anti-arthritic effect, and helps to control body weight, while leaf extracts have shown haematopoietic effect and the fruit extract had hepatoprotective effect [17]. *Olea europea* leaf extract and oleuropein were reported to be neuroprotective, reducing cell damage in Parkinson’s disease and oleuropein aglicone seems to prevent Alzheimer’s by inhibiting toxic amyloid aggregates in brain. Leaf and oil extracts were described to produce gastroprotective effect [35]. In addition, diarrhoea, haemorrhoids, rheumatism and asthma could also be prevented proving the significance of these fruit bioactive compounds in human disease prevention.

## Structure–Activity Relationship (SAR) Studies in Fruit Bioactive Natural Compounds with Respect to Human Diseases: Recent Updates

Fruits are abundant in different bioactive compounds including phytochemicals (phenolic acids, flavonoids, carotenoids, tannins, lignans and stilbenes), vitamins (provitamin A, C, E and K), minerals (potassium, calcium and magnesium) and dietary fibres which play a critical role in human health by alleviating several chronic diseases mainly coronary heart diseases, cancers, diabetes, cataracts and so on [49]. Though numerous reports describing the biological activities and compositions of fruit bioactive compounds have been published, there are limited studies relating to the structure activity relationships between the chemical compounds and their biological activity. With the advent of advanced technologies in genomic and proteomic levels, the SAR studies incorporating novel strategies would inevitably provide novel insights on several unexplored mechanisms. Bioactive compounds are particularly significant owing to their medical properties such as anti-inflammatory, antimicrobial, antioxidant, anticancer and their preventive effects against several chronic diseases. This section of the review describes the various SAR studies of fruit bioactive compounds with special emphasis on phytochemicals and their activities against human diseases, reported in recent years.

### Phenolic Acids

**Fig. 1:**
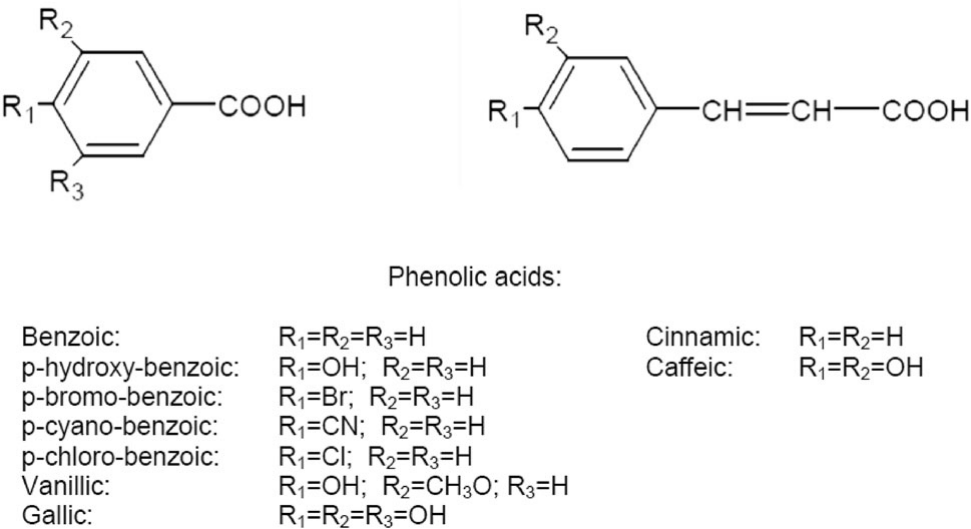
Basic chemical structures of phenolic acids

Phenolic acid compounds play a pivotal role as antioxidants and are the major antioxidant sources among plant foods which also can reduce the oxidative stress-induced tissue damage due to chronic diseases and anticancer activities. Several berries, apples, pears and grapes possess up to 200-300 mg polyphenols per 100 g fresh weight [50]. Phenolic acids have a carboxyl group linked to benzene ring (Fig. 1) and are classified into two major types based on their structure: benzoic acid derivatives (i.e. hydroxybenzoic acids, C6-C1) and cinnamic acid derivatives (i.e. hydroxycinnamic acids, C6-C3). Berries such as blueberries, raspberries and blackberries are rich in hydroxybenzoic and hydroxycinnamic acids. Some derivatives of hydroxybenzoic acids are currently used as additives to reduce the oxidation of nutrients and to enhance nutritional value in foods.

One of the early studies on structure–activity of phenolic acids and their derivatives stated that the hydroxycinnamic acid derivatives had higher antioxidant capacities when compared to their benzoic acid counterparts [51]. This ability was attributed to the presence of propenoic side chain, instead of the carboxylic group of benzoic acid derivatives; the conjugated double bond in their side chains has a profound effect by resonance on the phenoxyl radical, thus enhancing the antioxidant capacities.

Esterification process enhanced the solubility of phenolic acids in apolar media [52]. The authors synthesized protocatechuic acid (3,4-dihydroxybenzoic acid) and its esters; and upon evaluation found that the esterification has led to an enhanced radical scavenging ability and that the ester side chain is characteristic to the lipophilicity of the compounds without interfering with their inherent antioxidant properties.

Recently, the structure of gallic acid and its derivatives on their interaction with plant ferritin, a partially unstable molecule in the gastrointestinal tract was investigated [53]. The interaction was structure dependent with phenolic acids comprising three adjacent hydroxyl groups (gallic acid, methyl gallate and propylgallate) binding effectively with ferritin when compared to their two hydroxyl group containing analogues (protocatechuic acid and vanillic acid).

A set of 27 coumarin derivatives was synthesized and evaluated for their cytotoxicity on three human cancer lines and the results were promising [87]. The study postulated that 7,8- dihydroxy-4-methylcoumarins bearing alkyl groups at C3 position were the most effective subgroup. The second most active subgroup was 7,8- diacetoxy-4-methylcoumarins containing ethoxycarbonylmethyl and ethoxycarbonylethyl moieties at C3 position. These structure–activity relationship studies of methyl coumarins have identified potential candidates that can effectively target cancers. However, further studies are necessary to include these compounds in drugs.

### Flavonoids

**Fig. 2:**
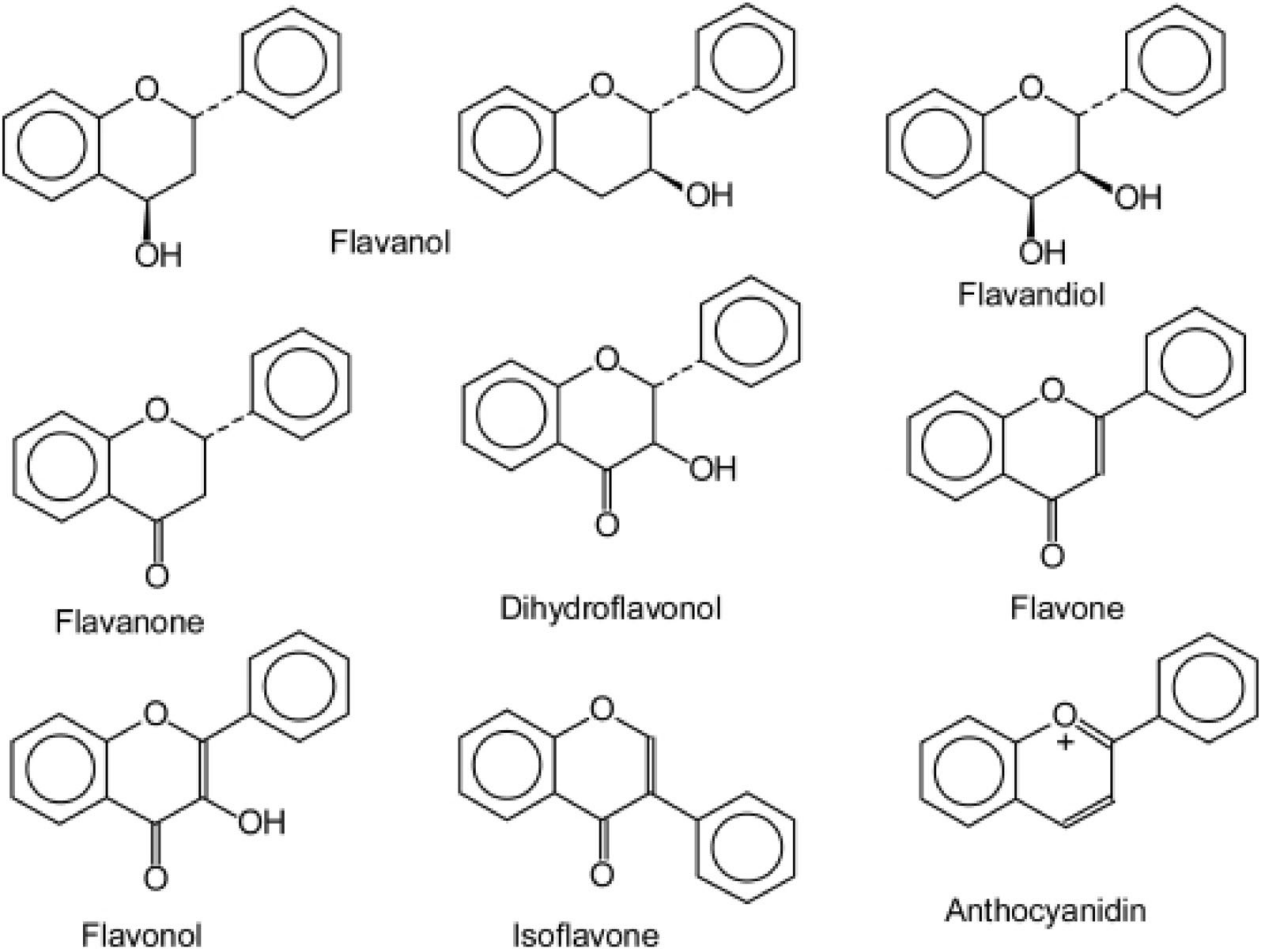
Basic chemical structures of flavonoids

Flavonoids, one of the largest groups of phenolic compounds studied extensively, have been known to play a significant role in various biological activities including antioxidant, antimicrobial, antimutagenic, cytotoxic and anticancer activities [54]. In fruits and vegetables, they are found in the form of glycosides or acylglycosides, while acylated, methylated and sulphate molecules are less frequent and in lower concentrations. Their core structure is a skeleton of diphenylpropane, namely, two benzene rings (A and B) linked by a three carbon chain that forms a closed heterocyclic pyran ring (C ring) with benzenic ring and hence referred as C6-C3-C6 (Fig. 2). They can be subdivided into flavonols (apples, blueberries), flavones, flavanones (citrus fruits), flavanonols, flavonols (grapes) or catechins and anthocyanins (berries and grapes) depending on the carbon of the C ring on which B ring is attached, and the degree of unsaturation and oxidation of the C ring (Fig. 2).

**Fig. 3:**
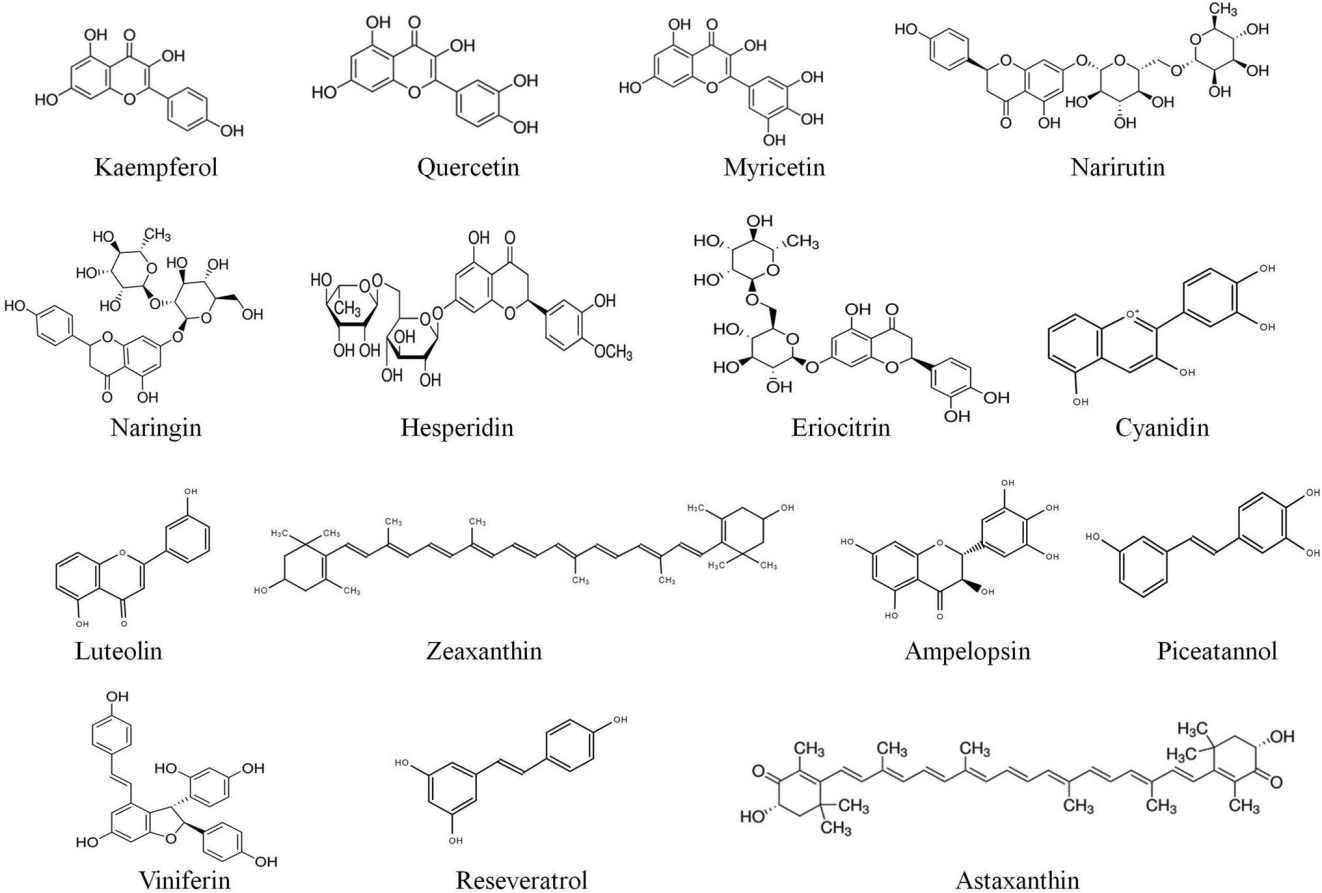
Chemical structures of major fruit bioactive compounds

Flavonols are a subclass of flavonoids that possess the 3-hydroxyflavone backbone and are typically featured with an unsaturated benzo-γ-pyrone (A and C Rings) displaced to a phenyl (B-ring) and as many as 7 hydroxyl groups surrounding their skeleton. Structure activity studies have determined that the number and position of hydroxyl groups are critical to their chemical structure and greatly affect their biological activities [55]. The chemical structures of major fruit bioactive compounds are given in Fig. 3.

One of the most studied flavonols compounds is kaempferol (KMF). Recently, a study proved that on treatment with kaempferol, myocardial ischemia–reperfusion injury in diabetic rats was greatly attenuated and that kaempferol pre-treatment significantly reduced hyperglycemia, maintained hemodynamic function and normalized oxidative stress [56]. Another report showed that kaempferol induced cellular apoptosis and may be a valuable adjuvant therapeutic agent in treatment of cervical cancer [57]. The multiple action mechanisms of kaempferol as an anticancer agent was extensively reviewed [58]. It acts on several intracellular as well as extracellular targets involved in cell signalling pathways that in turn are known to regulate the significant features of cancer growth progressions like apoptosis, cell cycle, invasion or metastasis, angiogenesis and inflammation. The understanding of mechanisms of action of KMF-mediated therapeutic effects will greatly aid the scientific community to design novel strategies for the treatment of dreadful diseases.

Quercetin, one of the most abundant plant flavonols, is active in several cancers, cardiovascular and neurodegenerative diseases. Its chemical modification has led to novel derivatives with improved biological effects, better bioavailability and antioxidant properties. Recently, a study investigated on O-substituted quercetin derivatives revealing monochloropivaloylquercetin and chloronaphthoquinonequercetin as potential therapeutics against chronic diseases like diabetes and neurodegenerative disorders [59]. Another interesting study suggested that quercetin therapy might improve heat stroke outcomes in rats by attenuating excessive hyperthermia as well as myocardial injury [60].

Myricetin, a plant flavonol abundant in fruits such as berries and grapes has been well known to act as effective therapeutic against diabetes mellitus [61]. A new study on mice revealed that myricetin ameliorated memory deficits thereby acting as an effective drug target for Alzheimer’s disease [62]. It was reported that myricetin induces apoptosis and enhanced cytotoxicity in ovarian cancer cell lines proving potential against ovarian cancer, the most lethal gynaecological cancer [63].

A latest structure–activity study illustrated that flavonoid derivatives having a hydroxyl group substituted at R-3 position on the C ring, have enhanced antioxidant activity. Flavone, having an OH group substituted at R-6 and R-7 position on ring A, showed similar antioxidant activity to flavone without -OH groups in the structure and slightly higher activity than the di-substituted flavone on the ring A [64]. The antioxidant properties of flavones are key determinants of their biological functions such as antimutagenic, anticancer and in delaying the ageing process.

Flavanones are abundant in citrus fruits and have a characteristic aglycone moiety which can combine with glycosides to form an array of flavanones. For instance, the most predominant flavanones in grapefruit are narirutin and naringin, hesperidin and narirutin in orange, and that in lemon is eriocitrin. It is interesting to note that narirutin and naringin have the same aglycone, naringenin, and hesperidin is the glycoside of hesperetin, while eriocitrin contains the aglycone eriodictyol. Very recently, the biochemical, pharmacological activities of Citrus flavanones was reviewed, highlighting the structure–function correlations and their ability to modulate signal cascades both in vitro and in vivo [6]. A meta-analysis of prospective cohort studies indicated that high intake of flavonoids is associated with reduced risk of mortality from cardiovascular diseases [65]. A recent study employed an integrated multi-target screening technique coupled with QSAR (quantitative structure–activity relationship) modeling revealing narirutin (abundant in oranges) as a potent agent that could be employed as therapeutic in Alzheimer’s disease [66]. The same research group previously demonstrated that hesperidin also exhibited similar multi-potent activity [67]. Another report states that naringenin; another citrus flavanone impairs dengue virus replication in human monocytes proving as a potential candidate for dengue treatments. Interestingly, a recent study employing quantum theory on the structure–activity relationship of 14 flavonoids from *Agrimonia pilosa* belonging to rosaceae, implied that glycosylation at C-6 could enhance antioxidant activity compared to the corresponding aglycones [68]. This study has shed insights on the quantum theory based principles which would aid in future research.

Anthocyanins, the pigmented flavonoids abundant in berries and grapes, are yet another interesting subclass of flavonoids widely known for their multifaceted medicinal traits. The various molecular mechanisms involved in the biological activities of anthocyanins were critically reviewed by analyzing the various signalling pathways regulating these health benefits [69]. The study also postulates the lack of synergistic or antagonistic effects of various anthocyanins in carcinogenesis, its initiation and progression which would inevitably generate new anthocyanin based anticancer drugs. A recent study described the pivotal multi-therapeutic role of anthocyanin compounds through various in vitro, in vivo and clinical trial experiments and their possible benefits against chronic diseases [70]. Another latest investigation on blueberry anthocyanins stated that they are readily metabolized to various phenolic acid derivatives thereby contributing extensively to human health [71]. A broad spectrum review on berry anthocyanins suggested a great correlation between berry anthocyanins intake and cardiovascular health reiterating the significance of these flavonoids [72].

Eight anthocyanidins, seven anthocyanins and two synthesized 4′-hydroxy flavyliums were evaluated and the results showed that most compounds had better activities than trolox and catechol [73]. The SAR study illustrated that, in the absence of the 3-OH group, radicals of the 4, 5 or 7-OH groups can only be stabilized by resonance through pyrylium oxygen, while 3-OH group improved hydrogen atom donation. Catechol structure enhanced both hydrogen and electron donation and those compounds lacking the catechol structure had a decreasing order of H-atom and electron donation consistent with decreasing number of their hydroxyl and/or methoxy groups. This study provided novel insights into antiradical and reductant activities of anthocyanins and anthocyanidins.

Recently, the molecular mechanism of action of a key anthocyanin, cyanidin was unveiled [74]. The authors performed a structure-based search for small molecules that inhibit signalling by the pro-inflammatory cytokine interleukin-17A and found that cyanidin specifically recognizes an IL-17A binding site in the IL-17A receptor subunit and inhibits their interaction thereby attenuating inflammation in mice. Their findings suggest the development of cyanidin into an effective small-molecule drug for the treatment of IL-17A–dependent inflammatory diseases and cancer.

### Carotenoids

Dietary carotenoids are tetraterpenoids primarily from fruits and vegetables and possess varied health benefits. α-carotene, β-carotene, γ-carotene, lycopene, and β-cryptoxanthin are precursors of vitamin A which also possess beneficial effects apart from antioxidant activity. Recent studies assessed three carotenoids- cryptocapsin, cryptocapsin-5,6-epoxide, and zeaxanthin among which cryptocapsin showed the highest bioactivity, while cryptocapsin-5,6-epoxide and zeaxanthin exhibited similar activity on anti-aggregation assays. The study was evident that cryptocapsin, cryptocapsin-5,6-epoxide, and zeaxanthin have anti-amyloidogenic potential and could be used for prevention and treatment of alzheimer’s disease [75]. Another xanthophyll carotenoid astaxanthin is proposed to be a preventive and therapeutic agent for cardiovascular diseases, one of the main contributors of worldwide mortality [76].

The pharmacological effect of the carotenoids Lutein and zeaxanthin on cognition diseases and visual disorders was reviewed extensively [77]. These carotenoids are protective against various diseases/disorders such as age-related macular degeneration (AMD), age-related cataract (ARC), ischemic/hypoxia induced retinopathy, retinal damage, retinitis pigmentosa, retinal detachment, uveitis and diabetic retinopathy. Properties such as physical blue light filtration and antioxidant activity are the major factors for their protective role. In addition to protection against light-induced oxidative damage, there are increasing evidences that they may also enhance normal ocular function by improving contrast sensitivity and reducing glare disability.

### Stilbenes

Stilbenes or stilbenoids are polyphenols that have gained importance in recent years. Grape berries are abundant in stilbenes with resveratrol present not only in the fruits but in leaves, stems, roots and canes too. They have been widely investigated owing to their antioxidant, antibacterial, antifungal, cardio protective, neuroprotective, anti-aging, and anticancer properties. About 13 Grapevine stilbenes were quantified with ε-viniferin and resveratrol being more abundant of all [78]. The research group also identified two potential inhibitors ampelopsin A and piceatannol that can be used in drugs against neurodegenerative diseases.

## Novel Strategies that Support Complete Utilization of Fruit Polyphenols

This section of the chapter reviews the various advanced strategies that are being employed in recent years in the field of natural bioactive compound research. In the diagnosis of neurodegenerative diseases, bioactive compounds from fruits have been promising by acting as potential inhibitors of amyloid aggregation. However, there is a lack of high-throughput screening methods for regular monitoring of amyloid aggregation in cells and that in vitro aggregation studies are not as effective in this context as they fail to mimic the cellular environment. Development of fast, reproducible in vivo and in vitro cell-based methods combined with in silico screening methods could represent a breakthrough for gaining insight into the molecular basis of amyloid aggregation as well as to identify drugs for diseases like Alzheimer’s.

The bioavailability of flavonoids is one of the major challenges faced by researchers. Several flavonoids which are of high medicinal value are present in limited quantities in nature. In addition, several fruit wastes are just discarded irrespective of their valuable polyphenol content. For instance, citrus fruit wastes and their by-products are rich in various bioactive compounds like pectin, antioxidants and essential oils. Recently, various novel and eco-friendly strategies for extraction of bioactive products from citrus wastes were reviewed [79]. The appropriate uses of these novel methods can inevitably enhance the bioavailability of flavonoid content for use in medicine and health.

Yet another challenge is the bio-accessibility of such natural bioactive compounds which is greatly affected by their chemical structure and matrix interaction. They have to be released from their matrix in order to be absorbable. Food processing can induce chemical or physical modifications in food that enhance bio-accessibility and bioavailability of phenolic compounds. They can a) modify the chemical structure and change into more bioaccessible and bioavailable forms; (ii) cleave covalent/hydrogen bonds/hydrophobic forces that attach phenolic compounds to the matrix; (c) damage structural barriers such as cell walls that prevent detachment and (d) incorporate microstructures that protect phenolic compounds until they are absorbed [80]. A thorough knowledge of food processing will thus be of great aid to enhance the bio-accessibility and bioavailability of bioactive phenolic compounds in fruits.

The advent of nanotechnology has enabled advanced drug delivery systems that can improve bioavailability of therapeutic agents. The use of solid lipid nanoparticle delivery systems and their efficiency over conventional systems as well as traditional systems like emulsions and polymer based delivery methods was reviewed [81]. Similarly, colloidal drug delivery systems like micro- and nanoemulsions, vesicular carriers and micro- and nanoparticles, loaded with natural active compounds also seem to be promising [82].

Molecular docking studies are rapidly gaining widespread popularity and employed to design structure based drugs as they can accurately predict the conformation of small-molecule ligands within the appropriate target binding site. Virtual screening and molecular docking of polyphenols can illustrate their binding ability to targets thereby generating efficient drug combinations. Recently, a study evaluated the α-amylase and α-glucosidase inhibitory activity of 26 polyphenols for treatment of diabetes and obesity using molecular docking [83]. Moreover, ligand based virtual screening methods offer high-throughput screening of polyphenols and have been routinely used in recent years.

Quantitative structure–activity relationship (QSAR) is one of the most important applications of chemometrics that provides useful information for the design of new compounds acting on a specific target. It attempts to find a consistent relationship between biological activity or toxicity and molecular properties. In other words, it enables identification of the response pharmacophore as well as the essential molecular fragments imparting antioxidant propensity to various classes of chemicals and serves as a reliable tool for searching efficient molecules with enhanced biological activity. Recently, a set of 29 flavonoids were tested for their inhibition against aldose reductase enzyme, a significant rate limiting enzyme in glucose metabolism by QSAR approach [84]. Another interesting study [85] employed comparative molecular field analysis (CoMFA) and comparative molecular similarity indices analysis (CoMSIA)-based QSAR methods to analyze flavonoid compounds and identified critical structures that could be modified to design potent inhibitory compounds.

Pharmacophore modeling is another novel technique that provides valuable information about the ligand-receptor interactions. They are probably one of the best options in finding chemical structures with therapeutically useful features. A pharmacophore model can be considered as the ensemble of steric and electrostatic features of different compounds that are required to ensure optimal supramolecular interactions with a specific biological target structure and to trigger or to block its biological response. This ability of a pharmacophore model is used to find new classes of inhibitors when one class is known, popularly called as “scaffold hopping”. A group of researchers employed pharmacophore modeling, QSAR and docking methods to search potential targets for the new flavonoid compounds they had synthesized against colon cancer cell lines [86]. Their studies deciphered the significance of these in silico tools in drug design.

## Concluding Remarks and Future Directions of Research

An effort has been made in this chapter to describe the recent updates on fruit bioactive compounds, their medicinal properties, the structure activity relationship of the compounds with relation to diseases and the novel strategies that aid in complete utilization of these natural compounds from fruits in order to develop novel drugs and therapies against human diseases. Several studies have made an effort to study the biological effects of the extracts but failed to identify the compound involved. Another challenge is that anticonvulsant and cardiovascular diseases were found only in few reports, while important neurodegenerative diseases like Parkinson and Alzheimer, and diseases of the modern society like obesity and depression needs further research.

There are still several gaps that need to be addressed. First, a broader framework has to materialize in order to understand the mechanisms by which polyphenols from fruits confer dietary health benefits to humans. Second, additional clinical studies are needed to determine their pharmacokinetic properties in human disease prevention. Third, appropriate biomarkers should be developed to determine the interactions between diseases and fruit polyphenols at cellular and molecular levels. Low bioavailability of certain flavonoids such as anthocyanins is a real challenge which necessitates further research. The advent of nanotechnology seems to be promising tool to enhance bioavailability of polyphenols in human systems. Advanced computational tools like molecular docking, QSAR studies, pharmacophore, highthroughput virtual screening techniques coupled with rigorous in vitro and in vivo assays would undoubtedly enable the efficient use of fruit bioactive compounds as therapeutic and nutraceutical agents in the near future.
